# Hemodynamic and mitochondrial effects of enalapril in experimental sepsis

**DOI:** 10.1186/cc11995

**Published:** 2013-03-19

**Authors:** AJ Pereira, V Jeger, T Corrêa, M Vuda, S Djafarzadeh, J Takala, S Jakob

**Affiliations:** 1 University Hospital (Inselspital)/University of Bern, Switzerland

## Introduction

Sepsis may cause mitochondrial dysfunction. The renin-angiotensin system (RAS) activity is increased in sepsis, and can interfere with the mitochondrial function either directly or by modifying hemodynamics. We studied the effects of ACE inhibition by enalapril on hemodynamics and hepatic mitochondrial function in sepsis. Two septic groups without enalapril and with different blood pressure targets served as controls.

## Methods

Sepsis (fecal peritonitis) was induced in 24 anesthetized, mechanically ventilated pigs (40.1 ± 2.1 kg), divided into three groups (*n *= 8 for each): ENL (enalapril pretreatment for 7 days and at 0.005 to 0.02 mg/kg/hour during the study; MAP target 75 to 85 mmHg), ML (target MAP 50 to 60 mmHg), and MH (target MAP 75 to 85 mmHg). Hemodynamic support with fluids, norepinephrine (NE; maximum dose 5,000 μg/hour), and antibiotics were started after 12 hours of peritonitis and continued for 48 hours.

## Results

All enalapril pigs received the maximum NE dose without reaching the target MAP. Enalapril resulted in lower MAP, higher CO, and transiently increased regional blood flows (Table 1). Fluid administration and urinary output were similar among groups. Liver mitochondrial respiration was reduced (State 3, State 4) in the enalapril group. One animal died in each ML and enalapril group.

**Table 1:** Hemodynamic variables, among times and groups

	BL	R0h	R12h	R24h	R36h	R48h	*P *(time×group)
MAP (ENL/MH/ML)	66 (9)/72 (15)/	65 (11)/73 (10)/	57 (4)/82 (6)/	58 (8)/78 (6)/	56 (12)/80 (5)/	56 (11)/79 (9)/	0.003
	65 (9)	81 (16)	72 (13)	67 (12)	61 (6)	56 (13)	
CO (ENL/MH/ML)	106 (13)/89 (7)/	92 (17)/67 (11)/	169 (16)/95 (19)/	187 (22)/129 (28)/	161 (25)/139 (32)/	157 (37)/140 (21)/	<0.0001
	105 (13)	88 (22)	104 (13)	143 (31)	145 (13)	150 (18)	
Carotid flow (ENL/MH/ML)	6 (2)/7 (2)/7 (2)	5 (1)/4 (1)/5 (2)	10 (3)/5 (1)/6 (2)	10 (3)/8 (1)/9 (2)	8 (2)/8 (2)/8 (2)	8 (3)/8 (2)/8 (2)	<0.0001
Femoral flow (ENL/MH/ML)	4 (1)/3 (1)/4 (2)	2 (1)/2 (0)/2 (1)	4 (1)/2 (1)/2 (0)	4 (1)/3 (1)/3 (1)	3 (1)/3 (1)/4 (1)	3 (2)/3 (1)/4 (1)	0.004

## Conclusion

Enalapril enhances cardiac output and early regional blood flows in sepsis despite lower MAP, and reduces liver mitochondrial respiration, suggesting that RAS is involved in the hemodynamic and metabolic changes in sepsis.

